# Bis{tris­[3-(2-pyrid­yl)-1*H*-pyrazole]nickel(II)} dodeca­molybdosilicate tetra­hydrate

**DOI:** 10.1107/S1600536810000978

**Published:** 2010-01-13

**Authors:** Xiutang Zhang, Dong Yuan, Peihai Wei, Bin Li, Bo Hu

**Affiliations:** a Institute of Advanced Materials Research, Department of Chemistry and Chemical Engineering, Shandong Institute of Education, Jinan 250013, People’s Republic of China; b College of Chemistry and Chemical Engineering, Liaocheng University, Liaocheng 252059, People’s Republic of China

## Abstract

The asymmetric unit of the title compound, [Ni(C_8_H_7_N_3_)_3_]_2_[SiMo_12_O_40_]·4H_2_O, consists of a complex [Ni(C_8_H_7_N_3_)_3_]^2+^ cation, half of a Keggin-type heteropolyanion [SiMo_12_O_40_]^4−^ and two uncoordinated water mol­ecules. The Ni^2+^ cation is surrounded in a slightly distorted octa­hedral coordination by six N atoms from three chelating 3-(2-pyrid­yl)-1*H*-pyrazole ligands. In the heteropolyanion, two O atoms of the central SiO_4_ group (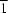 symmetry) are equally disordered about an inversion centre. N—H⋯O and O—H⋯O hydrogen bonding between the cations, anions and the uncoordinated water mol­ecules leads to a consolidation of the structure.

## Related literature

For general background to polyoxometalates, see: Pope & Müller (1991 ▶). For polyoxometalates modified with amines, see: Zhang, Dou *et al.* (2009 ▶); Zhang, Wei, Sun *et al.* (2009 ▶; Zhang, Wei, Shi *et al.* (2010 ▶). For the related structure of the Zn analogue that crystallizes as the hexa­hydrate, see: Zhang, Wei, Zhu *et al.* (2010 ▶). For a further related structure, see: Wu *et al.* (2003 ▶).
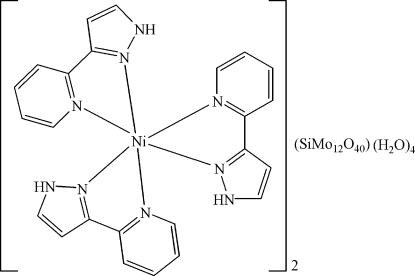


## Experimental

### 

#### Crystal data

[Ni(C_8_H_7_N_3_)_3_]_2_[SiMo_12_O_40_]·4H_2_O
*M* *_r_* = 2879.85
Monoclinic, 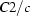

*a* = 18.687 (4) Å
*b* = 16.299 (3) Å
*c* = 27.604 (6) Å
β = 104.10 (3)°
*V* = 8154 (3) Å^3^
*Z* = 4
Mo *K*α radiation
μ = 2.35 mm^−1^
*T* = 293 K
0.12 × 0.10 × 0.08 mm

#### Data collection

Bruker APEXII CCD diffractometer
Absorption correction: multi-scan (*SADABS*; Bruker, 2001 ▶) *T*_min_ = 0.766, *T*_max_ = 0.835
28737 measured reflections
7188 independent reflections
3439 reflections with *I* > 2σ(*I*)
*R*_int_ = 0.125

#### Refinement

*R*[*F* ^2^ > 2σ(*F* ^2^)] = 0.056
*wR*(*F* ^2^) = 0.121
*S* = 1.00
7188 reflections
587 parameters
H-atom parameters constrained
Δρ_max_ = 1.78 e Å^−3^
Δρ_min_ = −0.66 e Å^−3^

### 

Data collection: *APEX2* (Bruker, 2004 ▶); cell refinement: *SAINT-Plus* (Bruker, 2001 ▶); data reduction: *SAINT-Plus*; program(s) used to solve structure: *SHELXS97* (Sheldrick, 2008 ▶); program(s) used to refine structure: *SHELXL97* (Sheldrick, 2008 ▶); molecular graphics: *SHELXTL* (Sheldrick, 2008 ▶); software used to prepare material for publication: *SHELXTL*.

## Supplementary Material

Crystal structure: contains datablocks global, I. DOI: 10.1107/S1600536810000978/wm2297sup1.cif

Structure factors: contains datablocks I. DOI: 10.1107/S1600536810000978/wm2297Isup2.hkl

Additional supplementary materials:  crystallographic information; 3D view; checkCIF report

## Figures and Tables

**Table 1:** Selected bond lengths (Å)

Ni1—N8	2.047 (11)
Ni1—N5	2.093 (11)
Ni1—N2	2.066 (11)
Ni1—N6	2.104 (10)
Ni1—N3	2.096 (12)
Ni1—N9	2.121 (11)
Si1—O18*A*	1.597 (13)
Si1—O18*B*	1.670 (13)
Si1—O1*A*	1.622 (14)
Si1—O1*B*	1.625 (13)

**Table 2:** Hydrogen-bond geometry (Å, °)

*D*—H⋯*A*	*D*—H	H⋯*A*	*D*⋯*A*	*D*—H⋯*A*
N1—H1*A*⋯O21^i^	0.86	2.01	2.850 (19)	165
N4—H4⋯O17^ii^	0.86	2.02	2.786 (13)	148
N7—H7*A*⋯O22^iii^	0.86	1.94	2.760 (16)	159

Symmetry codes: (i) 
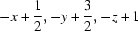
; (ii) 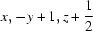; (iii) 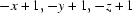.

**Fig. 1.**
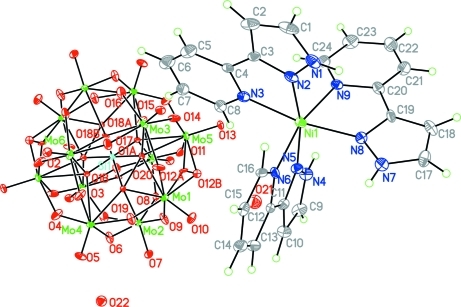
The building blocks of the title compound with the atom-labelling scheme. Displacement ellipsoids are drawn at the 30% probability level; H atoms are given as spheres of arbitrary radius. [Symmetry code: (A) -x+1/2, -y+1/2, -z.]

**Fig. 2.**
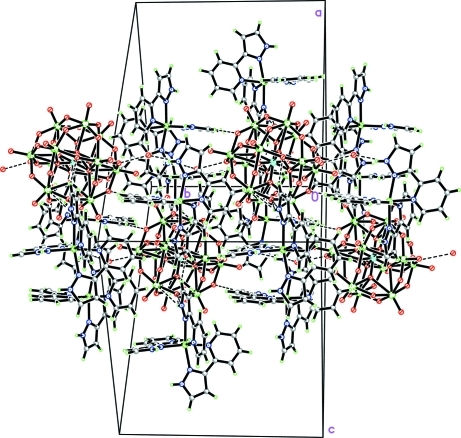
The crystal packing of the title compound, displayed with N—H···O and O—H···O hydrogen bonds as dashed lines.

## supplementary crystallographic information

### Comment

The design and synthesis of polyoxometalates has attracted continuous research interest not only because of their appealing structural and topological novelties, but also due to their interesting optical, electronic, magnetic, and catalytic properties, as well as their potential medical applications (Pope & Müller, 1991). In our research group, organic amines, such as 3-(2-pyridyl)pyrazole and pyrazine, are used to effectively modify polyoxomolybdates under hydrothermal condictions (Zhang, Dou *et al.*, 2009; Zhang, Wei, Sun *et al.*, 2009; Zhang, Wei, Shi *et al.*, 2010). Here, we describe the synthesis and structural characterization of the title compound.

As shown in Figure 1, the title compound consists of three subunits, *viz.* of a complex [Ni(C_8_H_7_N_3_)_3_]^2+^ cation, a [SiMo_12_O_40_]^4-^ heteropolyanion and of two uncoordinated water molecules. The nickel(II) ion is in a distorted octahedral coordination by six N atoms from three chelating 3-(2-pyridyl)pyrazole ligands. The Ni—N bond lengths are in the range of 2.063 (10)—2.131 (9) Å. In the Keggin-type heteropolyanion, each Mo atom is surrounded by six O atoms and the Si atom is located at the center of the anion. There are four kinds of O atoms present in the anion according to their coordination environments: O*_a_* (O atoms in the disordered SiO_4_ tetrahedron), O*_b_* (bridging O atoms between two triplet groups of MoO_6_ octahedra), O*_c_* (bridging O atoms within one triplet group of MoO_6_ octahedra) and O*_d_* (terminal O atoms). The Si—O bond distances are in the normal range of 1.571 (15)—1.635 (12) compared to reported distances in other dodecamolybdosilicates (Wu *et al.*, 2003; Zhang, Wei, Zhu *et al.*, 2010). The Mo—O bond distances vary widely from 1.627 (7) to 2.510 (15) Å. The shortest Mo—O bonds are in the range of 1.627 (7)—1.694 (7) Å for the terminal oxygen atoms. The longest Mo—O lengths are in the range of 2.2279 (14)—2.510 (15) Å for those oxygen atoms connected with both Mo and Si atoms.

N—H···O and O—H···O hydrogen bonding between the cationic and anionic moieties and the uncoordinated water molecules leads to a consolidation of the structure (Fig. 2; Table 2).

The TGA curve shows that the lattice water molecules and the organic ligands separate above *ca* 326 and 657 K, respectively. The overall thermal decomposition process can be described by the followed equation: C_48_H_50_Mo_12_N_18_Ni_2_O_44_Si + 81O_2_→ 25H_2_O + 48CO_2_ + 9N_2_O_5_ + 2NiO + SiO_2_ + 12MoO_3_.

### Experimental

A mixture of 3-(2-pyridyl)pyrazole (1 mmoL 0.14 g), sodium molybdate (2 mmoL, 0.48 g), sodium silicate nonahydrate (0.2 mmoL, 0.05 g) and nickel(II) chloride hexahydrate (0.25 mmol, 0.05 g) in 10 ml distilled water was sealed in a 25 ml Teflon-lined stainless steel autoclave and was kept at 433 K for three days. Green crystals suitable for the X-ray experiment were obtained. Anal. C_48_H_50_Mo_12_N_18_Ni_2_O_44_Si: C, 20.02; H, 1.75; N, 8.75%. Found: C, 19.28; H, 2.07; N, 8.43%. IR(cm^-1^): 3376, 3136, 2961, 1614, 1568, 1522, 1457, 1439, 1364, 1300, 1097, 950, 913, 812, 636, 507.

### Refinement

All hydrogen atoms bound to aromatic carbon atoms were refined in calculated positions using a riding model with a C—H distance of 0.93 Å and *U*_iso_ = 1.2*U*_eq_(C). Hydrogen atoms attached to aromatic N atoms were refined with a N—H distance of 0.86 Å and *U*_iso_ = 1.2*U*_eq_(N). The hydrogen atoms of the two uncoordinated water molecules could not be located unambiguously from difference Fourier maps, probably due to disorder of the water molecules. Thus the structure was refined without the H atoms of the water molecules (which includes the water O atoms O21, O22). In the SiO_4_ unit, the two oxygen atoms (O1 and O18) are equally disordered about the inversion centre. One of the bridging O atoms (O12) is also disordered and was refined with split positions and an occupancy ratio of 1:1. In the final difference Fourier map the highest peak is 2.87 Å from atom O22 and the deepest hole is 0.11 Å from atom O8. The highest peak is located in the voids of the crystal structure and may be associated with an additional water molecule. However, refinement of this position did not result in a reasonable model. Hence this position was also excluded from the final refinement.

### Crystal data

**Table:**

[Ni(C_8_H_7_N_3_)_3_]_2_[SiMo_12_O_40_]·4H_2_O	*F*(000) = 5560
*M**_r_* = 2879.85	*D*_x_ = 2.346 Mg m^−^^3^
Monoclinic, *C*2/*c*	Mo *K*α radiation, λ = 0.71073 Å
Hall symbol: -C 2yc	Cell parameters from 7031 reflections
*a* = 18.687 (4) Å	θ = 1.7–25.0°
*b* = 16.299 (3) Å	µ = 2.35 mm^−^^1^
*c* = 27.604 (6) Å	*T* = 293 K
β = 104.10 (3)°	Block, green
*V* = 8154 (3) Å^3^	0.12 × 0.10 × 0.08 mm
*Z* = 4	

### Data collection

**Table:**

Bruker APEXII CCD diffractometer	7188 independent reflections
Radiation source: fine-focus sealed tube	3439 reflections with *I* > 2σ(*I*)
graphite	*R*_int_ = 0.125
phi and ω scans	θ_max_ = 25.0°, θ_min_ = 1.5°
Absorption correction: multi-scan (*SADABS*; Bruker, 2001)	*h* = −22→22
*T*_min_ = 0.766, *T*_max_ = 0.835	*k* = −19→19
28737 measured reflections	*l* = −32→32

### Refinement

**Table:**

Refinement on *F*^2^	Primary atom site location: structure-invariant direct methods
Least-squares matrix: full	Secondary atom site location: difference Fourier map
*R*[*F*^2^ > 2σ(*F*^2^)] = 0.056	Hydrogen site location: inferred from neighbouring sites
*wR*(*F*^2^) = 0.121	H-atom parameters constrained
*S* = 1.00	*w* = 1/[σ^2^(*F*_o_^2^) + (0.020*P*)^2^] where *P* = (*F*_o_^2^ + 2*F*_c_^2^)/3
7188 reflections	(Δ/σ)_max_ < 0.001
587 parameters	Δρ_max_ = 1.78 e Å^−^^3^
0 restraints	Δρ_min_ = −0.66 e Å^−^^3^

### Special details

**Table:**

Geometry. All esds (except the esd in the dihedral angle between two l.s. planes) are estimated using the full covariance matrix. The cell esds are taken into account individually in the estimation of esds in distances, angles and torsion angles; correlations between esds in cell parameters are only used when they are defined by crystal symmetry. An approximate (isotropic) treatment of cell esds is used for estimating esds involving l.s. planes.
Refinement. Refinement of *F*^2^ against ALL reflections. The weighted *R*-factor *wR* and goodness of fit *S* are based on *F*^2^, conventional *R*-factors *R* are based on *F*, with *F* set to zero for negative *F*^2^. The threshold expression of *F*^2^ > σ(*F*^2^) is used only for calculating *R*-factors(gt) *etc*. and is not relevant to the choice of reflections for refinement. *R*-factors based on *F*^2^ are statistically about twice as large as those based on *F*, and *R*- factors based on ALL data will be even larger.

### Fractional atomic coordinates and isotropic or equivalent isotropic displacement parameters (Å^2^)

**Table:**

	*x*	*y*	*z*	*U*_iso_*/*U*_eq_	Occ. (<1)
Mo1	0.35240 (6)	0.39842 (7)	0.08084 (4)	0.0443 (3)	
Mo2	0.41863 (6)	0.32246 (7)	−0.01978 (4)	0.0435 (3)	
Mo3	0.17276 (7)	0.34378 (8)	0.09029 (4)	0.0473 (4)	
Mo4	0.41827 (6)	0.19458 (8)	0.08355 (4)	0.0465 (4)	
Mo5	0.19742 (7)	0.45683 (7)	−0.01322 (5)	0.0504 (4)	
Mo6	0.24752 (7)	0.14446 (8)	0.11101 (4)	0.0518 (4)	
Ni1	0.20687 (9)	0.68505 (11)	0.80894 (6)	0.0483 (5)	
Si1	0.2500	0.2500	0.0000	0.0298 (12)	
C1	0.0571 (10)	0.6492 (14)	0.9038 (7)	0.094 (7)	
H1	0.0339	0.6609	0.9292	0.112*	
C2	0.0465 (9)	0.5804 (13)	0.8729 (7)	0.091 (7)	
H2	0.0143	0.5370	0.8733	0.110*	
C3	0.0961 (8)	0.5903 (10)	0.8402 (6)	0.054 (4)	
C4	0.1117 (8)	0.5414 (11)	0.8024 (6)	0.054 (4)	
C5	0.0818 (9)	0.4649 (14)	0.7921 (7)	0.087 (6)	
H5	0.0509	0.4437	0.8108	0.105*	
C6	0.0967 (10)	0.4219 (13)	0.7560 (8)	0.098 (7)	
H6	0.0761	0.3701	0.7487	0.117*	
C7	0.1423 (9)	0.4526 (11)	0.7292 (6)	0.068 (5)	
H7	0.1521	0.4238	0.7025	0.082*	
C8	0.1740 (8)	0.5294 (10)	0.7432 (5)	0.060 (4)	
H8	0.2075	0.5497	0.7262	0.072*	
C9	0.3644 (10)	0.6954 (10)	0.7209 (5)	0.075 (5)	
H9	0.3831	0.7078	0.6935	0.090*	
C10	0.4029 (8)	0.6601 (10)	0.7658 (6)	0.077 (5)	
H10	0.4520	0.6436	0.7745	0.092*	
C11	0.3521 (8)	0.6553 (8)	0.7941 (5)	0.049 (4)	
C12	0.3576 (7)	0.6248 (7)	0.8444 (5)	0.038 (3)	
C13	0.4223 (8)	0.5908 (9)	0.8744 (6)	0.068 (5)	
H13	0.4653	0.5878	0.8633	0.081*	
C14	0.4201 (8)	0.5628 (9)	0.9197 (6)	0.069 (5)	
H14	0.4623	0.5394	0.9401	0.083*	
C15	0.3569 (8)	0.5679 (8)	0.9368 (5)	0.051 (4)	
H15	0.3562	0.5480	0.9682	0.061*	
C16	0.2960 (7)	0.6023 (8)	0.9071 (5)	0.045 (4)	
H16	0.2533	0.6064	0.9186	0.054*	
C17	0.2717 (10)	0.9142 (11)	0.8778 (6)	0.077 (5)	
H17	0.2973	0.9505	0.9019	0.092*	
C18	0.2178 (9)	0.9341 (10)	0.8373 (6)	0.077 (5)	
H18	0.1991	0.9863	0.8282	0.093*	
C19	0.1958 (8)	0.8617 (9)	0.8119 (6)	0.057 (4)	
C20	0.1435 (8)	0.8442 (11)	0.7662 (5)	0.059 (4)	
C21	0.1056 (9)	0.9007 (10)	0.7363 (6)	0.082 (6)	
H21	0.1121	0.9556	0.7454	0.098*	
C22	0.0578 (10)	0.8808 (12)	0.6928 (8)	0.103 (7)	
H22	0.0308	0.9203	0.6717	0.124*	
C23	0.0516 (9)	0.8002 (14)	0.6820 (6)	0.095 (6)	
H23	0.0210	0.7830	0.6519	0.114*	
C24	0.0910 (8)	0.7415 (9)	0.7157 (6)	0.067 (5)	
H24	0.0837	0.6861	0.7082	0.081*	
N1	0.1099 (7)	0.6972 (8)	0.8887 (5)	0.076 (4)	
H1A	0.1262	0.7435	0.9018	0.091*	
N2	0.1317 (6)	0.6609 (8)	0.8510 (4)	0.057 (3)	
N3	0.1596 (6)	0.5741 (7)	0.7786 (4)	0.055 (3)	
N4	0.2975 (7)	0.7076 (7)	0.7250 (4)	0.063 (4)	
H4	0.2633	0.7290	0.7019	0.075*	
N5	0.2877 (6)	0.6839 (7)	0.7681 (4)	0.055 (3)	
N6	0.2958 (6)	0.6311 (6)	0.8608 (4)	0.044 (3)	
N7	0.2818 (6)	0.8333 (8)	0.8773 (4)	0.066 (4)	
H7A	0.3136	0.8063	0.8993	0.080*	
N8	0.2349 (6)	0.7999 (7)	0.8373 (4)	0.052 (3)	
N9	0.1365 (6)	0.7627 (8)	0.7565 (4)	0.050 (3)	
O1A	0.2482 (7)	0.3343 (8)	0.0309 (5)	0.027 (4)	0.50
O1B	0.2934 (7)	0.1816 (9)	0.0391 (5)	0.029 (4)	0.50
O2	0.2429 (5)	0.0914 (5)	0.1612 (3)	0.060 (3)	
O3	0.3450 (5)	0.1717 (6)	0.1221 (3)	0.084 (3)	
O4	0.3951 (5)	0.0981 (6)	0.0507 (4)	0.090 (3)	
O5	0.4948 (4)	0.1706 (6)	0.1251 (3)	0.072 (3)	
O6	0.4537 (5)	0.2423 (5)	0.0337 (3)	0.065 (3)	
O7	0.5002 (5)	0.3493 (5)	−0.0291 (3)	0.064 (3)	
O8	0.3538 (5)	0.3703 (5)	−0.0718 (3)	0.083 (3)	
O9	0.4062 (5)	0.3955 (5)	0.0265 (3)	0.069 (3)	
O10	0.4013 (5)	0.4725 (5)	0.1145 (3)	0.068 (3)	
O11	0.2353 (5)	0.4438 (6)	−0.0677 (4)	0.087 (3)	
O12A	0.2849 (9)	0.4501 (13)	0.0254 (7)	0.043 (6)	0.50
O12B	0.2927 (14)	0.4877 (14)	0.0385 (9)	0.057 (9)*	0.50
O13	0.1700 (5)	0.5541 (5)	−0.0185 (3)	0.064 (3)	
O14	0.1565 (4)	0.4247 (6)	0.0429 (3)	0.079 (3)	
O15	0.1428 (4)	0.3897 (5)	0.1359 (3)	0.061 (3)	
O16	0.0955 (6)	0.2809 (6)	0.0605 (4)	0.097 (4)	
O17	0.2229 (5)	0.2482 (6)	0.1281 (4)	0.090 (3)	
O18A	0.1691 (8)	0.2190 (8)	−0.0272 (5)	0.029 (4)	0.50
O18B	0.1992 (7)	0.2358 (9)	0.0414 (5)	0.031 (4)	0.50
O19	0.4037 (5)	0.3075 (5)	0.1058 (3)	0.067 (3)	
O20	0.2782 (4)	0.3836 (6)	0.1090 (3)	0.079 (3)	
O21	0.3627 (7)	0.6467 (9)	0.0636 (5)	0.157 (5)	
O22	0.6160 (6)	0.2176 (6)	0.0381 (4)	0.096 (4)	

### Atomic displacement parameters (Å^2^)

**Table:**

	*U*^11^	*U*^22^	*U*^33^	*U*^12^	*U*^13^	*U*^23^
Mo1	0.0396 (8)	0.0504 (8)	0.0407 (8)	−0.0109 (6)	0.0055 (6)	−0.0107 (7)
Mo2	0.0335 (7)	0.0450 (8)	0.0510 (8)	−0.0066 (6)	0.0085 (6)	−0.0027 (7)
Mo3	0.0542 (9)	0.0530 (9)	0.0335 (7)	0.0146 (7)	0.0085 (6)	−0.0077 (7)
Mo4	0.0344 (7)	0.0582 (9)	0.0412 (8)	−0.0001 (6)	−0.0016 (6)	0.0041 (7)
Mo5	0.0592 (9)	0.0338 (7)	0.0639 (9)	0.0095 (7)	0.0260 (7)	0.0070 (7)
Mo6	0.0765 (10)	0.0469 (8)	0.0300 (7)	−0.0085 (7)	0.0094 (7)	0.0062 (6)
Ni1	0.0458 (11)	0.0548 (13)	0.0445 (11)	0.0074 (10)	0.0111 (9)	0.0094 (10)
Si1	0.031 (3)	0.032 (3)	0.022 (3)	0.001 (2)	−0.001 (2)	−0.001 (2)
C1	0.062 (13)	0.16 (2)	0.071 (14)	0.042 (14)	0.031 (11)	0.054 (14)
C2	0.050 (12)	0.14 (2)	0.074 (14)	0.007 (12)	−0.007 (11)	0.057 (13)
C3	0.035 (7)	0.063 (8)	0.058 (8)	0.000 (7)	0.001 (6)	0.021 (7)
C4	0.033 (9)	0.058 (12)	0.059 (11)	0.009 (8)	−0.011 (8)	0.002 (10)
C5	0.071 (13)	0.114 (19)	0.063 (14)	−0.017 (13)	−0.011 (11)	0.029 (13)
C6	0.078 (15)	0.112 (19)	0.084 (16)	−0.020 (13)	−0.017 (13)	0.009 (14)
C7	0.063 (12)	0.072 (13)	0.062 (12)	0.001 (10)	−0.001 (9)	0.005 (10)
C8	0.064 (11)	0.065 (12)	0.044 (10)	0.004 (10)	0.002 (9)	0.015 (9)
C9	0.080 (13)	0.110 (15)	0.040 (10)	−0.019 (12)	0.023 (10)	0.008 (10)
C10	0.063 (12)	0.092 (14)	0.077 (13)	0.005 (10)	0.020 (10)	0.003 (11)
C11	0.053 (10)	0.055 (10)	0.046 (10)	−0.010 (8)	0.023 (8)	−0.002 (8)
C12	0.038 (9)	0.034 (9)	0.042 (9)	−0.002 (7)	0.008 (7)	−0.002 (7)
C13	0.051 (11)	0.081 (13)	0.069 (12)	0.000 (9)	0.010 (9)	0.015 (10)
C14	0.061 (12)	0.078 (12)	0.054 (11)	−0.008 (9)	−0.015 (9)	0.006 (10)
C15	0.077 (12)	0.045 (9)	0.031 (9)	−0.014 (9)	0.012 (9)	0.008 (7)
C16	0.029 (8)	0.056 (10)	0.050 (9)	−0.002 (7)	0.009 (7)	0.015 (8)
C17	0.115 (16)	0.067 (13)	0.051 (12)	−0.015 (12)	0.024 (11)	−0.022 (10)
C18	0.095 (14)	0.072 (14)	0.066 (13)	0.036 (11)	0.023 (11)	0.009 (11)
C19	0.069 (12)	0.034 (10)	0.077 (13)	0.009 (9)	0.035 (10)	−0.004 (10)
C20	0.067 (11)	0.064 (12)	0.039 (10)	0.016 (10)	0.002 (8)	0.006 (9)
C21	0.080 (13)	0.070 (13)	0.076 (13)	−0.001 (11)	−0.019 (10)	0.019 (11)
C22	0.094 (16)	0.077 (15)	0.122 (19)	0.015 (13)	−0.006 (13)	0.029 (14)
C23	0.082 (14)	0.128 (19)	0.053 (12)	−0.002 (14)	−0.027 (10)	0.005 (13)
C24	0.063 (12)	0.066 (12)	0.067 (12)	0.001 (9)	0.005 (9)	0.013 (10)
N1	0.070 (10)	0.086 (11)	0.064 (10)	0.027 (8)	0.002 (8)	0.003 (9)
N2	0.050 (8)	0.078 (10)	0.041 (8)	0.021 (7)	0.011 (6)	−0.002 (7)
N3	0.048 (8)	0.070 (10)	0.047 (8)	0.006 (7)	0.014 (7)	0.001 (7)
N4	0.071 (10)	0.083 (10)	0.035 (8)	0.002 (8)	0.014 (7)	0.020 (7)
N5	0.048 (8)	0.064 (9)	0.049 (8)	−0.001 (7)	0.003 (7)	0.013 (7)
N6	0.049 (8)	0.032 (7)	0.051 (8)	0.009 (5)	0.014 (6)	0.010 (6)
N7	0.069 (9)	0.073 (10)	0.056 (9)	0.005 (8)	0.014 (7)	0.002 (8)
N8	0.058 (8)	0.057 (9)	0.042 (8)	−0.001 (7)	0.015 (6)	−0.008 (7)
N9	0.040 (8)	0.064 (9)	0.041 (8)	0.007 (7)	0.002 (6)	−0.003 (7)
O1A	0.032 (7)	0.031 (7)	0.020 (7)	0.002 (6)	0.008 (6)	0.003 (6)
O1B	0.022 (7)	0.036 (8)	0.029 (7)	−0.001 (6)	0.006 (6)	0.006 (6)
O2	0.063 (6)	0.070 (7)	0.043 (6)	−0.003 (5)	0.002 (5)	0.023 (5)
O3	0.089 (7)	0.087 (7)	0.092 (7)	−0.045 (5)	0.051 (5)	−0.039 (5)
O4	0.094 (7)	0.101 (7)	0.088 (7)	−0.046 (6)	0.050 (5)	−0.038 (6)
O5	0.049 (6)	0.105 (9)	0.050 (6)	0.027 (6)	−0.009 (5)	−0.002 (6)
O6	0.111 (9)	0.049 (6)	0.047 (6)	0.023 (6)	0.042 (6)	0.010 (5)
O7	0.060 (7)	0.063 (7)	0.077 (7)	−0.012 (5)	0.033 (5)	0.006 (6)
O8	0.088 (6)	0.041 (5)	0.087 (6)	0.010 (5)	−0.046 (5)	−0.010 (5)
O9	0.108 (8)	0.063 (7)	0.052 (6)	0.044 (6)	0.051 (6)	0.026 (5)
O10	0.094 (8)	0.063 (7)	0.047 (6)	−0.031 (6)	0.019 (5)	−0.023 (5)
O11	0.080 (6)	0.101 (7)	0.093 (7)	−0.047 (5)	0.049 (5)	−0.054 (6)
O12A	0.012 (10)	0.064 (17)	0.047 (14)	−0.011 (11)	−0.002 (9)	0.013 (13)
O13	0.065 (7)	0.034 (6)	0.089 (8)	0.013 (5)	0.008 (6)	0.006 (5)
O14	0.040 (6)	0.131 (10)	0.067 (7)	0.003 (6)	0.015 (5)	0.049 (7)
O15	0.056 (6)	0.078 (7)	0.054 (6)	0.004 (5)	0.025 (5)	−0.014 (5)
O16	0.104 (7)	0.066 (6)	0.085 (7)	0.006 (6)	−0.048 (5)	−0.005 (5)
O17	0.091 (7)	0.053 (6)	0.094 (7)	0.013 (5)	−0.037 (5)	−0.011 (5)
O18A	0.032 (10)	0.027 (10)	0.025 (9)	0.002 (8)	0.000 (8)	0.010 (7)
O18B	0.014 (8)	0.040 (11)	0.043 (11)	0.010 (7)	0.015 (8)	0.000 (8)
O19	0.106 (8)	0.056 (7)	0.054 (6)	0.025 (6)	0.048 (6)	0.010 (5)
O20	0.031 (6)	0.132 (10)	0.073 (7)	0.012 (6)	0.011 (5)	0.059 (7)
O21	0.157 (5)	0.157 (5)	0.157 (5)	−0.0005 (11)	0.0390 (16)	−0.0004 (11)
O22	0.096 (4)	0.096 (4)	0.096 (4)	0.0005 (10)	0.0228 (13)	−0.0002 (11)

### Geometric parameters (Å, °)

**Table:**

Mo1—O10	1.657 (8)	C3—C4	1.397 (18)
Mo1—O20	1.763 (8)	C4—N3	1.344 (17)
Mo1—O19	1.808 (8)	C4—C5	1.37 (2)
Mo1—O12A	1.924 (18)	C5—C6	1.30 (2)
Mo1—O9	2.000 (8)	C5—H5	0.9300
Mo1—O12B	2.02 (3)	C6—C7	1.35 (2)
Mo1—O1A	2.340 (13)	C6—H6	0.9300
Mo1—O18A^i^	2.394 (14)	C7—C8	1.398 (18)
Mo2—O7	1.666 (8)	C7—H7	0.9300
Mo2—O9	1.802 (8)	C8—N3	1.298 (16)
Mo2—O8	1.813 (8)	C8—H8	0.9300
Mo2—O6	1.961 (8)	C9—N4	1.298 (16)
Mo2—O16^i^	2.006 (9)	C9—C10	1.395 (18)
Mo2—O18B^i^	2.337 (13)	C9—H9	0.9300
Mo2—O18A^i^	2.420 (14)	C10—C11	1.372 (17)
Mo3—O15	1.673 (8)	C10—H10	0.9300
Mo3—O16	1.798 (9)	C11—N5	1.326 (15)
Mo3—O14	1.830 (9)	C11—C12	1.455 (16)
Mo3—O17	1.979 (9)	C12—N6	1.342 (14)
Mo3—O20	2.019 (8)	C12—C13	1.404 (16)
Mo3—O18B	2.343 (14)	C13—C14	1.340 (17)
Mo3—O1A	2.414 (13)	C13—H13	0.9300
Mo4—O5	1.647 (8)	C14—C15	1.376 (17)
Mo4—O4	1.814 (9)	C14—H14	0.9300
Mo4—O6	1.838 (8)	C15—C16	1.351 (16)
Mo4—O3	1.964 (9)	C15—H15	0.9300
Mo4—O19	1.981 (9)	C16—N6	1.359 (14)
Mo4—O1B	2.366 (13)	C16—H16	0.9300
Mo4—O18A^i^	2.415 (13)	C17—N7	1.332 (17)
Mo5—O13	1.662 (8)	C17—C18	1.352 (18)
Mo5—O12A	1.724 (17)	C17—H17	0.9300
Mo5—O11	1.824 (9)	C18—C19	1.383 (18)
Mo5—O14	1.958 (8)	C18—H18	0.9300
Mo5—O4^i^	1.997 (9)	C19—N8	1.337 (16)
Mo5—O12B	2.06 (2)	C19—C20	1.426 (18)
Mo5—O1B^i^	2.385 (14)	C20—C21	1.321 (18)
Mo5—O1A	2.411 (13)	C20—N9	1.355 (17)
Mo6—O2	1.653 (8)	C21—C22	1.35 (2)
Mo6—O17	1.844 (9)	C21—H21	0.9300
Mo6—O3	1.827 (9)	C22—C23	1.35 (2)
Mo6—O11^i^	1.946 (9)	C22—H22	0.9300
Mo6—O8^i^	1.954 (9)	C23—C24	1.410 (19)
Mo6—O1B	2.422 (13)	C23—H23	0.9300
Mo6—O18B	2.426 (14)	C24—N9	1.282 (15)
Ni1—N8	2.047 (11)	C24—H24	0.9300
Ni1—N5	2.093 (11)	N1—N2	1.345 (14)
Ni1—N2	2.066 (11)	N1—H1A	0.8600
Ni1—N6	2.104 (10)	N4—N5	1.305 (13)
Ni1—N3	2.096 (12)	N4—H4	0.8600
Ni1—N9	2.121 (11)	N7—N8	1.345 (13)
Si1—O18A	1.597 (13)	N7—H7A	0.8600
Si1—O18A^i^	1.597 (13)	O1A—O18A^i^	1.798 (18)
Si1—O18B^i^	1.670 (13)	O1B—Mo5^i^	2.385 (14)
Si1—O18B	1.670 (13)	O4—Mo5^i^	1.997 (9)
Si1—O1A^i^	1.622 (14)	O8—Mo6^i^	1.954 (9)
Si1—O1A	1.622 (14)	O11—Mo6^i^	1.946 (9)
Si1—O1B^i^	1.625 (13)	O12A—O12B	0.71 (3)
Si1—O1B	1.625 (13)	O16—Mo2^i^	2.006 (9)
C1—N1	1.399 (19)	O18A—O18B	1.858 (19)
C1—C2	1.39 (2)	O18A—O1A^i^	1.798 (18)
C1—H1	0.9300	O18A—Mo2^i^	2.420 (14)
C2—C3	1.45 (2)	O18A—Mo1^i^	2.394 (14)
C2—H2	0.9300	O18A—Mo4^i^	2.415 (13)
C3—N2	1.327 (16)	O18B—Mo2^i^	2.337 (13)
			
O10—Mo1—O20	103.7 (4)	O18A—Si1—O1B^i^	69.1 (6)
O10—Mo1—O19	102.4 (4)	O18A^i^—Si1—O1B^i^	110.9 (6)
O20—Mo1—O19	96.9 (4)	O18B^i^—Si1—O1B^i^	74.1 (6)
O10—Mo1—O12A	107.0 (7)	O18B—Si1—O1B^i^	105.9 (6)
O20—Mo1—O12A	88.7 (6)	O1A^i^—Si1—O1B^i^	107.6 (7)
O19—Mo1—O12A	147.9 (7)	O1A—Si1—O1B^i^	72.4 (7)
O10—Mo1—O9	97.4 (4)	O18A—Si1—O1B	110.9 (6)
O20—Mo1—O9	157.0 (4)	O18A^i^—Si1—O1B	69.1 (6)
O19—Mo1—O9	87.2 (3)	O18B^i^—Si1—O1B	105.9 (6)
O12A—Mo1—O9	76.5 (6)	O18B—Si1—O1B	74.1 (6)
O10—Mo1—O12B	87.2 (7)	O1A^i^—Si1—O1B	72.4 (7)
O20—Mo1—O12B	88.3 (8)	O1A—Si1—O1B	107.6 (7)
O19—Mo1—O12B	167.6 (7)	O1B^i^—Si1—O1B	180.0 (11)
O12A—Mo1—O12B	20.5 (8)	N1—C1—C2	105.9 (17)
O9—Mo1—O12B	83.7 (8)	N1—C1—H1	127.0
O10—Mo1—O1A	157.3 (5)	C2—C1—H1	127.0
O20—Mo1—O1A	64.4 (4)	C1—C2—C3	105.9 (18)
O19—Mo1—O1A	98.4 (5)	C1—C2—H2	127.1
O12A—Mo1—O1A	55.7 (7)	C3—C2—H2	127.1
O9—Mo1—O1A	92.6 (4)	N2—C3—C4	118.6 (16)
O12B—Mo1—O1A	73.7 (7)	N2—C3—C2	108.4 (16)
O10—Mo1—O18A^i^	156.5 (5)	C4—C3—C2	133.0 (18)
O20—Mo1—O18A^i^	97.6 (5)	N3—C4—C5	123.1 (16)
O19—Mo1—O18A^i^	64.8 (4)	N3—C4—C3	115.1 (16)
O12A—Mo1—O18A^i^	83.1 (7)	C5—C4—C3	121.8 (19)
O9—Mo1—O18A^i^	63.6 (4)	C6—C5—C4	120 (2)
O12B—Mo1—O18A^i^	103.4 (7)	C6—C5—H5	120.1
O1A—Mo1—O18A^i^	44.6 (4)	C4—C5—H5	120.1
O7—Mo2—O9	102.5 (4)	C5—C6—C7	120 (2)
O7—Mo2—O8	103.0 (5)	C5—C6—H6	120.0
O9—Mo2—O8	95.8 (4)	C7—C6—H6	120.0
O7—Mo2—O6	98.1 (4)	C6—C7—C8	117.4 (17)
O9—Mo2—O6	89.1 (3)	C6—C7—H7	121.3
O8—Mo2—O6	156.8 (4)	C8—C7—H7	121.3
O7—Mo2—O16^i^	97.7 (4)	N3—C8—C7	123.9 (15)
O9—Mo2—O16^i^	158.4 (4)	N3—C8—H8	118.0
O8—Mo2—O16^i^	86.9 (4)	C7—C8—H8	118.0
O6—Mo2—O16^i^	80.6 (4)	N4—C9—C10	106.6 (14)
O7—Mo2—O18B^i^	155.2 (5)	N4—C9—H9	126.7
O9—Mo2—O18B^i^	100.0 (5)	C10—C9—H9	126.7
O8—Mo2—O18B^i^	64.3 (5)	C11—C10—C9	104.5 (14)
O6—Mo2—O18B^i^	92.4 (4)	C11—C10—H10	127.8
O16^i^—Mo2—O18B^i^	61.9 (4)	C9—C10—H10	127.8
O7—Mo2—O18A^i^	157.1 (4)	N5—C11—C10	109.6 (13)
O9—Mo2—O18A^i^	65.4 (4)	N5—C11—C12	118.7 (13)
O8—Mo2—O18A^i^	97.8 (5)	C10—C11—C12	131.8 (15)
O6—Mo2—O18A^i^	63.6 (4)	N6—C12—C13	121.3 (13)
O16^i^—Mo2—O18A^i^	92.9 (5)	N6—C12—C11	115.5 (12)
O18B^i^—Mo2—O18A^i^	45.9 (4)	C13—C12—C11	123.3 (14)
O15—Mo3—O16	102.8 (5)	C14—C13—C12	117.9 (15)
O15—Mo3—O14	100.8 (4)	C14—C13—H13	121.1
O16—Mo3—O14	96.3 (4)	C12—C13—H13	121.1
O15—Mo3—O17	99.0 (4)	C13—C14—C15	121.5 (15)
O16—Mo3—O17	91.0 (4)	C13—C14—H14	119.3
O14—Mo3—O17	156.8 (4)	C15—C14—H14	119.3
O15—Mo3—O20	98.5 (4)	C16—C15—C14	118.9 (13)
O16—Mo3—O20	157.7 (5)	C16—C15—H15	120.5
O14—Mo3—O20	86.1 (4)	C14—C15—H15	120.5
O17—Mo3—O20	79.1 (4)	C15—C16—N6	121.5 (12)
O15—Mo3—O18B	157.7 (5)	C15—C16—H16	119.3
O16—Mo3—O18B	64.3 (5)	N6—C16—H16	119.3
O14—Mo3—O18B	98.7 (5)	N7—C17—C18	107.9 (15)
O17—Mo3—O18B	64.8 (4)	N7—C17—H17	126.1
O20—Mo3—O18B	93.4 (4)	C18—C17—H17	126.1
O15—Mo3—O1A	153.8 (4)	C17—C18—C19	106.4 (15)
O16—Mo3—O1A	100.9 (5)	C17—C18—H18	126.8
O14—Mo3—O1A	65.4 (4)	C19—C18—H18	126.8
O17—Mo3—O1A	91.6 (5)	N8—C19—C20	118.9 (15)
O20—Mo3—O1A	59.9 (4)	N8—C19—C18	108.7 (15)
O18B—Mo3—O1A	47.2 (5)	C20—C19—C18	132.3 (16)
O5—Mo4—O4	101.8 (5)	C21—C20—N9	123.3 (15)
O5—Mo4—O6	102.2 (4)	C21—C20—C19	124.2 (17)
O4—Mo4—O6	94.5 (4)	N9—C20—C19	112.5 (13)
O5—Mo4—O3	100.3 (4)	C20—C21—C22	121.8 (18)
O4—Mo4—O3	89.6 (4)	C20—C21—H21	119.1
O6—Mo4—O3	155.7 (4)	C22—C21—H21	119.1
O5—Mo4—O19	99.5 (4)	C23—C22—C21	115.7 (18)
O4—Mo4—O19	157.9 (4)	C23—C22—H22	122.1
O6—Mo4—O19	86.6 (3)	C21—C22—H22	122.1
O3—Mo4—O19	80.9 (4)	C22—C23—C24	120.8 (17)
O5—Mo4—O1B	156.7 (5)	C22—C23—H23	119.6
O4—Mo4—O1B	65.0 (5)	C24—C23—H23	119.6
O6—Mo4—O1B	98.0 (4)	N9—C24—C23	121.7 (16)
O3—Mo4—O1B	62.2 (4)	N9—C24—H24	119.2
O19—Mo4—O1B	93.0 (4)	C23—C24—H24	119.2
O5—Mo4—O18A^i^	157.3 (5)	N2—N1—C1	110.7 (15)
O4—Mo4—O18A^i^	98.1 (5)	N2—N1—H1A	124.7
O6—Mo4—O18A^i^	65.2 (4)	C1—N1—H1A	124.7
O3—Mo4—O18A^i^	90.5 (5)	N1—N2—C3	109.0 (14)
O19—Mo4—O18A^i^	62.4 (4)	N1—N2—Ni1	136.7 (12)
O1B—Mo4—O18A^i^	44.9 (5)	C3—N2—Ni1	114.3 (11)
O13—Mo5—O12A	109.9 (8)	C8—N3—C4	115.6 (14)
O13—Mo5—O11	102.2 (5)	C8—N3—Ni1	129.8 (12)
O12A—Mo5—O11	90.2 (7)	C4—N3—Ni1	114.4 (11)
O13—Mo5—O14	98.8 (4)	C9—N4—N5	112.7 (12)
O12A—Mo5—O14	89.3 (7)	C9—N4—H4	123.7
O11—Mo5—O14	157.8 (4)	N5—N4—H4	123.7
O13—Mo5—O4^i^	99.9 (4)	C11—N5—N4	106.7 (12)
O12A—Mo5—O4^i^	149.6 (8)	C11—N5—Ni1	113.0 (10)
O11—Mo5—O4^i^	88.9 (4)	N4—N5—Ni1	140.1 (10)
O14—Mo5—O4^i^	80.5 (4)	C12—N6—C16	119.0 (11)
O13—Mo5—O12B	91.4 (7)	C12—N6—Ni1	113.8 (9)
O12A—Mo5—O12B	19.1 (10)	C16—N6—Ni1	127.2 (9)
O11—Mo5—O12B	99.0 (8)	C17—N7—N8	110.1 (13)
O14—Mo5—O12B	87.4 (8)	C17—N7—H7A	124.9
O4^i^—Mo5—O12B	164.5 (8)	N8—N7—H7A	124.9
O13—Mo5—O1B^i^	156.8 (4)	N7—N8—C19	106.8 (12)
O12A—Mo5—O1B^i^	90.0 (8)	N7—N8—Ni1	137.6 (11)
O11—Mo5—O1B^i^	64.7 (4)	C19—N8—Ni1	115.6 (11)
O14—Mo5—O1B^i^	93.1 (4)	C24—N9—C20	116.6 (13)
O4^i^—Mo5—O1B^i^	62.4 (4)	C24—N9—Ni1	127.4 (11)
O12B—Mo5—O1B^i^	109.0 (7)	C20—N9—Ni1	115.8 (9)
O13—Mo5—O1A	155.5 (4)	Si1—O1A—O18A^i^	55.4 (6)
O12A—Mo5—O1A	55.8 (8)	Si1—O1A—Mo1	124.6 (7)
O11—Mo5—O1A	97.8 (5)	O18A^i^—O1A—Mo1	69.3 (6)
O14—Mo5—O1A	63.9 (4)	Si1—O1A—Mo5	119.5 (7)
O4^i^—Mo5—O1A	94.3 (4)	O18A^i^—O1A—Mo5	127.6 (8)
O12B—Mo5—O1A	71.6 (7)	Mo1—O1A—Mo5	95.5 (5)
O1B^i^—Mo5—O1A	47.1 (4)	Si1—O1A—Mo3	120.1 (7)
O2—Mo6—O17	101.4 (5)	O18A^i^—O1A—Mo3	136.3 (8)
O2—Mo6—O3	103.9 (4)	Mo1—O1A—Mo3	96.4 (5)
O17—Mo6—O3	92.0 (4)	Mo5—O1A—Mo3	93.8 (5)
O2—Mo6—O11^i^	100.3 (4)	Si1—O1B—Mo4	123.5 (7)
O17—Mo6—O11^i^	157.5 (4)	Si1—O1B—Mo5^i^	120.7 (7)
O3—Mo6—O11^i^	88.6 (4)	Mo4—O1B—Mo5^i^	96.1 (5)
O2—Mo6—O8^i^	99.1 (4)	Si1—O1B—Mo6	119.8 (7)
O17—Mo6—O8^i^	89.2 (4)	Mo4—O1B—Mo6	95.7 (5)
O3—Mo6—O8^i^	156.2 (4)	Mo5^i^—O1B—Mo6	94.3 (5)
O11^i^—Mo6—O8^i^	81.5 (4)	Mo6—O3—Mo4	138.9 (5)
O2—Mo6—O1B	156.7 (4)	Mo4—O4—Mo5^i^	136.0 (6)
O17—Mo6—O1B	98.1 (5)	Mo4—O6—Mo2	136.9 (5)
O3—Mo6—O1B	62.5 (4)	Mo2—O8—Mo6^i^	139.1 (6)
O11^i^—Mo6—O1B	62.4 (4)	Mo2—O9—Mo1	136.3 (5)
O8^i^—Mo6—O1B	93.8 (4)	Mo5—O11—Mo6^i^	138.4 (6)
O2—Mo6—O18B	154.1 (4)	O12B—O12A—Mo5	108 (3)
O17—Mo6—O18B	64.7 (4)	O12B—O12A—Mo1	88 (3)
O3—Mo6—O18B	98.5 (5)	Mo5—O12A—Mo1	149.2 (11)
O11^i^—Mo6—O18B	93.0 (5)	O12A—O12B—Mo5	53 (3)
O8^i^—Mo6—O18B	60.8 (4)	O12A—O12B—Mo1	72 (3)
O1B—Mo6—O18B	48.4 (4)	Mo5—O12B—Mo1	119.2 (11)
N8—Ni1—N5	93.6 (4)	Mo3—O14—Mo5	136.8 (5)
N8—Ni1—N2	96.0 (5)	Mo3—O16—Mo2^i^	135.8 (6)
N5—Ni1—N2	168.3 (5)	Mo6—O17—Mo3	134.6 (5)
N8—Ni1—N6	92.0 (4)	Si1—O18A—O18B	57.2 (6)
N5—Ni1—N6	79.0 (4)	Si1—O18A—O1A^i^	56.7 (6)
N2—Ni1—N6	94.0 (4)	O18B—O18A—O1A^i^	94.5 (8)
N8—Ni1—N3	170.2 (5)	Si1—O18A—Mo2^i^	121.6 (7)
N5—Ni1—N3	93.8 (5)	O18B—O18A—Mo2^i^	64.7 (6)
N2—Ni1—N3	77.4 (5)	O1A^i^—O18A—Mo2^i^	126.8 (8)
N6—Ni1—N3	95.6 (4)	Si1—O18A—Mo1^i^	122.7 (7)
N8—Ni1—N9	77.0 (5)	O18B—O18A—Mo1^i^	135.4 (8)
N5—Ni1—N9	92.6 (4)	O1A^i^—O18A—Mo1^i^	66.1 (6)
N2—Ni1—N9	96.0 (4)	Mo2^i^—O18A—Mo1^i^	94.3 (5)
N6—Ni1—N9	165.8 (4)	Si1—O18A—Mo4^i^	122.2 (7)
N3—Ni1—N9	96.3 (5)	O18B—O18A—Mo4^i^	124.0 (8)
O18A—Si1—O18A^i^	180.0 (15)	O1A^i^—O18A—Mo4^i^	134.5 (8)
O18A—Si1—O18B^i^	110.7 (7)	Mo2^i^—O18A—Mo4^i^	93.9 (5)
O18A^i^—Si1—O18B^i^	69.3 (7)	Mo1^i^—O18A—Mo4^i^	94.7 (5)
O18A—Si1—O18B	69.3 (7)	Si1—O18B—O18A	53.5 (6)
O18A^i^—Si1—O18B	110.7 (7)	Si1—O18B—Mo2^i^	122.6 (7)
O18B^i^—Si1—O18B	180.0 (12)	O18A—O18B—Mo2^i^	69.4 (6)
O18A—Si1—O1A^i^	67.9 (7)	Si1—O18B—Mo3	121.7 (7)
O18A^i^—Si1—O1A^i^	112.1 (7)	O18A—O18B—Mo3	130.1 (8)
O18B^i^—Si1—O1A^i^	70.7 (7)	Mo2^i^—O18B—Mo3	97.7 (5)
O18B—Si1—O1A^i^	109.3 (7)	Si1—O18B—Mo6	117.6 (6)
O18A—Si1—O1A	112.1 (7)	O18A—O18B—Mo6	132.7 (8)
O18A^i^—Si1—O1A	67.9 (7)	Mo2^i^—O18B—Mo6	95.6 (5)
O18B^i^—Si1—O1A	109.3 (7)	Mo3—O18B—Mo6	95.4 (5)
O18B—Si1—O1A	70.7 (7)	Mo1—O19—Mo4	138.0 (5)
O1A^i^—Si1—O1A	180.0 (12)	Mo1—O20—Mo3	139.0 (5)

Symmetry codes: (i) −*x*+1/2, −*y*+1/2, −*z*.

### Hydrogen-bond geometry (Å, °)

**Table:**

*D*—H···*A*	*D*—H	H···*A*	*D*···*A*	*D*—H···*A*
N1—H1A···O21^ii^	0.86	2.01	2.850 (19)	165
N4—H4···O17^iii^	0.86	2.02	2.786 (13)	148
N7—H7A···O22^iv^	0.86	1.94	2.760 (16)	159

Symmetry codes: (ii) −*x*+1/2, −*y*+3/2, −*z*+1; (iii) *x*, −*y*+1, *z*+1/2; (iv) −*x*+1, −*y*+1, −*z*+1.
